# Feline pemphigus foliaceus: original case series and a comprehensive literature review

**DOI:** 10.1186/s12917-018-1739-y

**Published:** 2019-01-09

**Authors:** Petra Bizikova, Amanda Burrows

**Affiliations:** 1 Department of Clinical Sciences, College of Veterinary Medicine, North Carolina State University, 1060 William Moore Drive, Raleigh, NC 27607 USA; 2 Center for Comparative Medicine and Translational Research, North Carolina State University, Raleigh, NC USA; 3 Murdoch University Veterinary Hospital, School of Veterinary and Biomedical Science, Murdoch University, Murdoch, WA Australia

**Keywords:** Auto-immune skin diseases, Auto-immunity, Feline, Cat, Dermatology, Pemphigus, Skin

## Abstract

**Background:**

Since the first description of feline pemphigus foliaceus (PF) more than 30 years ago, numerous case reports have been published, while larger case series have remained rare. This large body of information, if extrapolated, could address clinical discrepancies and expand our knowledge about the treatment of feline PF.

This manuscript reviews cases of feline PF published between 1950 and 2016 and adds additional 35 original cases to provide further insight into the clinical aspect and treatment outcome of this disease.

**Results:**

Feline PF, while being a primary acantholytic pustular dermatosis, presents most often with crusts and erosions that predominantly affect the face and feet. More than half of cats with active disease exhibits non-dermatological signs such as lethargy, fever and/or anorexia. The prognosis of feline PF is good as the majority of cats rapidly achieve disease control even with the most basic treatment such as glucocorticoid monotherapy. Most PF-affected cats, however, require long-term treatment and, like other autoimmune diseases, feline PF has a tendency to relapse spontaneously or with treatment changes.

**Conclusions:**

Therefore, despite the overall good prognosis cats with PF can be given, owners should be informed and prepared for these circumstances, which may reduce the risk of euthanasia in the case of disease relapse, and improve treatment compliance.

## Background

Pemphigus foliaceus (PF) is the most common autoimmune skin disease recognized in cats [1, 2]. It is defined as a pustular erosive and crusting dermatosis commonly involving the face, ears and feet [3]. In addition, variable frequencies of involvement of the periareolar (2–20%) and claw folds (30–90%), as well as generalized distribution have been reported [1, 4–8].

The diagnostic approach to feline PF has not changed in over 30 years in veterinary medicine, and it is still based on clinical and microscopic confirmation of a subcorneal pustular dermatitis (i.e. presence of pustules, secondary superficial erosions and crusts) with acantholysis. Indeed, the list of diseases presenting with primary subcorneal pustules with acantholysis in cats is limited to PF, and to anecdotal reports of pustular dermatophytosis; the latter has been reported to exhibit minimal to no acantholysis [9]. Bullous impetigo, a subcorneal pustular dermatitis with variable degree of acantholysis caused by *Staphylococcus aureus* and *pseudintermedius* in people and dogs, has not been well characterised in cats [10–12]. Immunological testing for antikeratinocyte autoantibodies by direct or indirect immunofluorescence is neither commercially available for cats, nor is the sensitivity and, particularly, specificity of such tests known for feline PF.

Various treatment modalities have been published over the years for feline PF. Glucocorticoids are the most frequently selected drugs despite reports of their variable efficacy ranging from 35 to 97% [8, 13–15]. Chlorambucil, aurothioglucose (gold salts), ciclosporin and even azathioprine have been used when glucocorticoids failed to provide adequate control, or when cats were unable to tolerate prolonged glucocorticoid therapy due to concurrent health issues [4, 6, 7, 13, 14]. Due to the current unavailability of the original formulation of aurothioglucose and the sensitivity of cats to azathioprine [16, 17], chlorambucil and ciclosporin remain the most commonly recommended adjunctive drugs for the management of feline PF [3, 15]. Nonetheless, the evidence of efficacy for these drugs to induce disease control and to maintain it is limited [6, 7].

There are only few large case series of feline PF published [1, 6–8], but many individual case reports can be found. This large body of information, if extrapolated, could address discrepancies in clinical presentation and expand our knowledge about the treatment of cats with PF. As such, the goal of this study was to perform a comprehensive review of all cases of feline PF published between 1950 and 2016 with a focus on clinical aspects and treatment outcomes. A retrospective analysis of additional 35 cats with PF from both authors’ institutions was performed and expanded the data available for analysis.

## Methods

### Original case series

Cats included in this report were selected from cases diagnosed and treated at the authors’ institutions between January 2000 and June 2017 using following criteria: i) clinical evidence of superficial pustules and/or secondary erosions and/or crusts; ii) lack of response to appropriate antibiotic treatment (of at least 3-week duration); iii) presence of numerous acantholytic cells on cytology and/or histopathology, and iv) a follow-up of at least 3 months. There was no age restriction. Information about the signalment, lesion distribution, presence of systemic signs, treatment outcome and the time of follow-up was extracted and recorded in a tabular form. If disease control (DC) was achieved, the time to DC and the drug(s) given at the time of DC were recorded. Disease control was defined as a time at which new lesions ceased to form and established lesions (pustules, erosions and crusts overlying active erosions) had mostly or fully healed. When compared to the human PF outcome measures, the DC definition used here would more correspond with “the end of consolidation phase” timing in human PF [18]. Finally, information about the treatment discontinuation and relapse episodes was noted.

### Comprehensive literature review

A literature search for any study detailing clinical and/or treatment evaluation of feline PF cases published between 1950 and 2016 was conducted using four databases: Pubmed (pubmed.gov), Web of Science (Thomson Reuters), CAB Abstracts (EBSCOhost Research Databases) and CAB Abstracts Archive (EBSCOhost Research Databases). Reviews that did not include clinical cases were excluded. The following search strategy was used for all databases:

- (pemphigus OR autoimmune skin OR auto-immune skin) AND (cat OR cats OR feline OR felines) NOT (human OR humans OR child* OR patient*)

There was no date or language restriction placed on the manuscript search. Additionally, the bibliographies of all selected articles and published abstracts from annual meetings of the European Society of Veterinary Dermatology/European College of Veterinary Dermatology, American Academy of Veterinary Dermatology/American College of Veterinary Dermatology and World Congresses of Veterinary Dermatology between 1995 and 2016 were screened for additional reports.

**Fig. 1:**
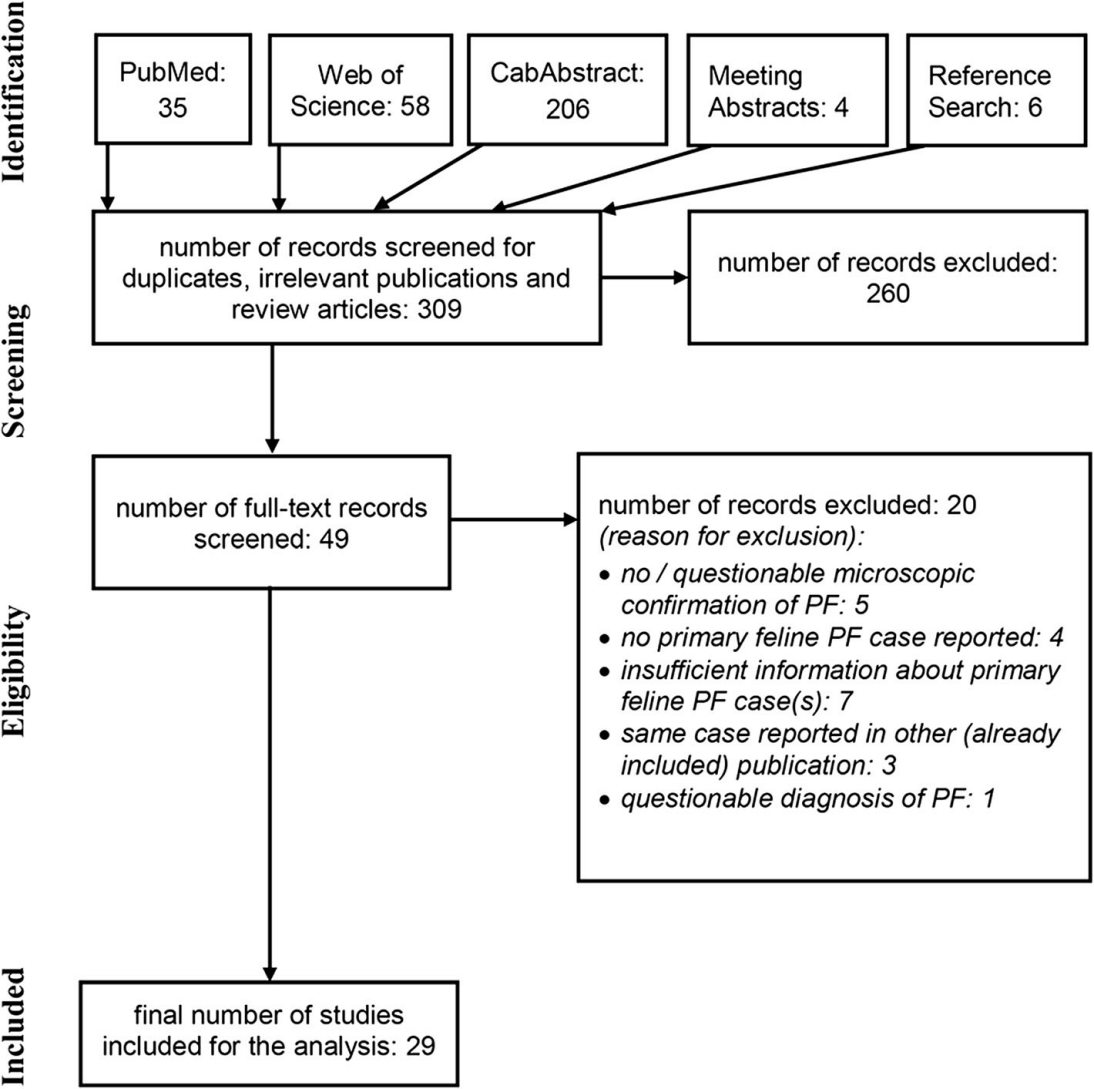
A flow chart diagram depicting the performed literature search. Only publications in which the author(s) described superficial pustular and/or erosive and/or crusting dermatitis with microscopic confirmation of acantholysis were included. Review articles and publications not containing primary PF cases or containing cases already published elsewhere, or publications with cases of unclear etiology (not fulfilling the clinical and microscopic criteria listed above) were excluded. Excluded publications are referenced [74–93]

Only publications in which the author(s) described superficial pustular and/or erosive and/or crusting dermatitis with microscopic confirmation of acantholysis were included. Review articles and publications not containing primary PF cases or containing cases already published elsewhere, or publications with cases of unclear etiology (not fulfilling the clinical and microscopic criteria listed above) were excluded (Fig. 1). There was no age restriction set for the included cats. No specific length of follow-up was required.

Data regarding the signalment, clinical features (lesion distribution, presence of systemic signs) and treatment outcome (achievement of DC, time to DC, drugs at the time of DC) were extracted and presented in a tabular form by PB. Missing data for incompletely described (or photographically documented) cases were marked as “not reported” and accounted for in the percentage calculations. The lesion symmetry was recorded using both the authors’ description and images from the publications. The data were verified by the second author (MB) and any discrepancies were resolved by consensus.

### Statistical analyses

The comparison of independent categorical parameters of continuous values was made using Mann-Whitney U test. The threshold of significance was set at *P* = *0.05*. Statistic analyses were made using Prism 7 (Graphpad software, San Diego, CA, USA).

## Results

### Signalment and clinical features

#### Original case series

Thirty-five cats met the inclusion criteria. Acantholysis was confirmed in all cases by cytological evaluation, and in most cases by histological evaluation (24/35; 69%). Most cats were middle aged at the onset of their disease (median (mean): 6 (6.8) years; range: 0.4–15 years), and female cats were marginally over-represented (female-to-male ratio of 1.7). Cats affected with PF belonged to variety of different breeds including Domestic short-haired cats (19/35; 54%), Siamese cats (3/35; 9%), Domestic medium-haired cats (2/35; 6%), Ragdoll cats (2/35; 6%), and one of each of the following breeds (Birman, British short-haired, Burmese, Cornish rex, Himalayan, Napoleon, Russian blue, Tonkinese and Turkish Van cats). A specific trigger was not confirmed for any cat, although a regular vaccination closely preceded the onset of the PF in two cats (6%). In one of these cats, a long-term complete remission off drugs without relapses was reported (follow up: 55 months).

**Fig. 2:**
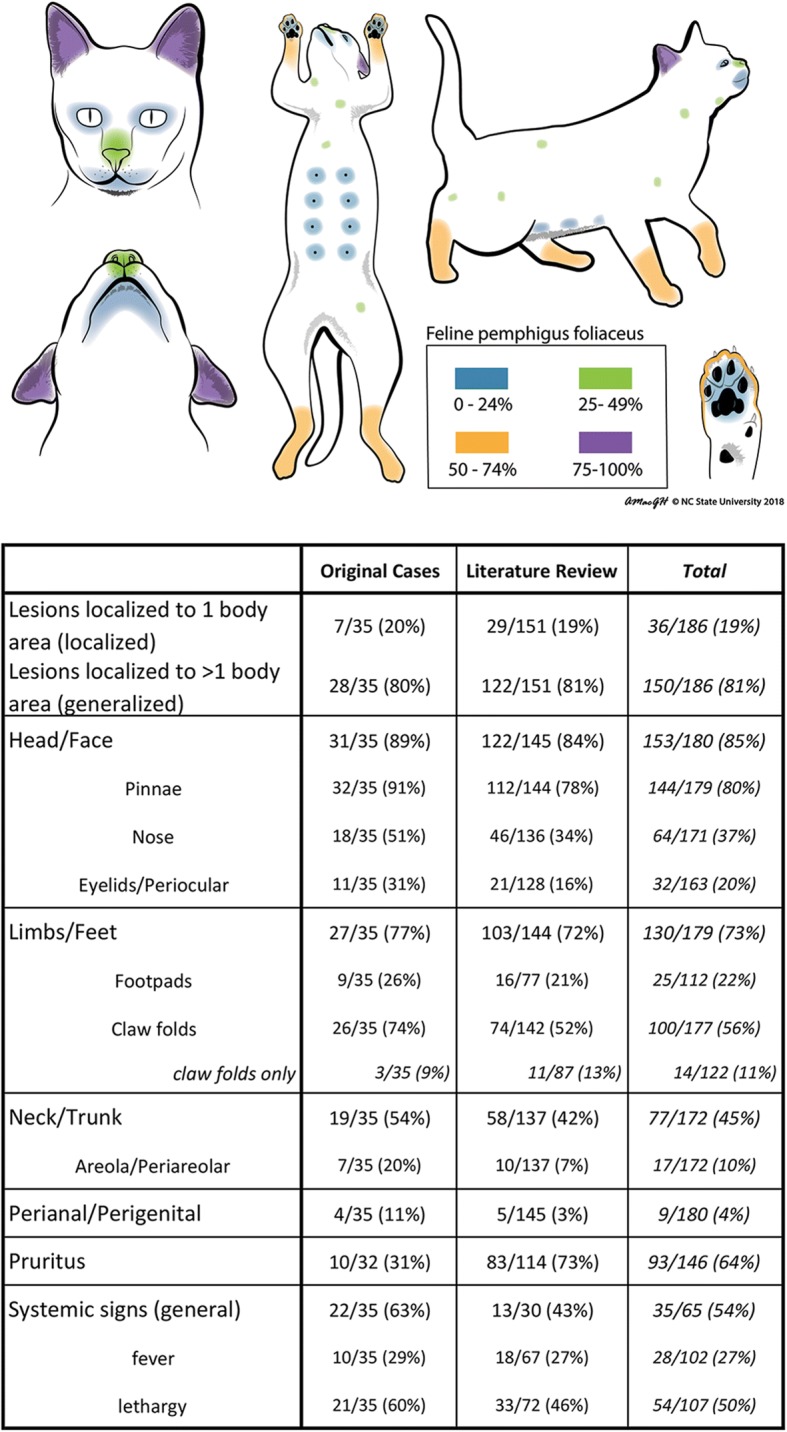
Feline pemphigus foliaceus lesion distribution diagram and individual data of lesion distribution (based on the original cases and the literature review)

**Fig. 3:**
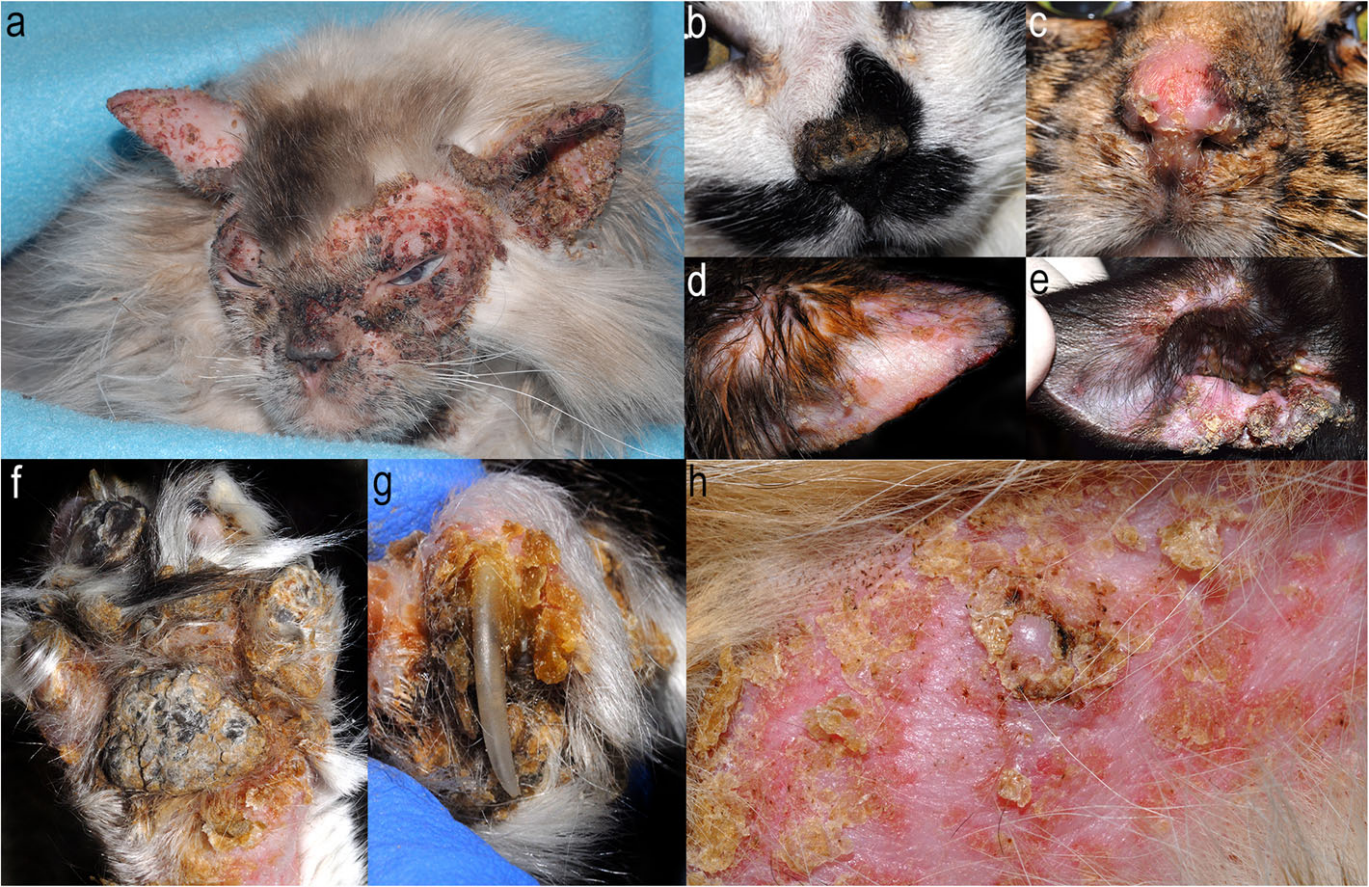
Clinical photos depicting characteristic skin lesions and their distribution. **a** multifocal pinpoint to coalescing erosions and crusts on the face and pinnae; **b**, **c** nasal planum erosions and crusts; **d**, **e** erosion and crusts on convex and concave pinnae; **f** thick crusting and hyperkeratosis on a pawpad; **g** thick crusting and purulent exudation affecting the nail fold; **h** multifocal erosions and crusts near the areolar region. Acknowledgements for clinical photographs: Michael Rossi (**a**), Aurore Laprais (**b**), Marcy Murphy (**d**)

Skin lesions in PF-affected cats consisted of pustules, erosions and/or crusts as expected based on the inclusion criteria. They were symmetric in the majority of cats (33/34; 97%) for which this information was available, and usually affected two or more body regions (28/35; 80%). The most commonly affected body regions were the face/head (31/35; 89%) and limbs (27/35; 77%); the most commonly affected skin sites were the pinnae (32/35; 91%) and claw folds (26/35; 74%) (Figs. 2 and 3). Pruritus status was reported in 32 cats, 10 of which were pruritic. The degree of pruritus was noted in seven of the ten cats (mild: 3, moderate: 2, severe: 2). Systemic signs were recorded in 22 of 35 cats (63%); 21 of 35 cats (60%) were lethargic, and 10 of 35 cats (29%) were febrile.

#### Comprehensive literature review

The literature search is summarized in Fig. 1. Twenty-nine studies reporting cats with PF were selected [1, 4–8, 13, 19–40]. Twenty-one publications reported a single case, two reported two to five cases, three reported 6 to 10 cases and three reported 11 to 57 cases. Twenty-six publications were journal articles (14 in English, six in French, two in Japanese, two in Portuguese and one in Danish languages), three were abstracts (in English) and one was a thesis (in Portuguese).

A total of 162 cats with PF were included for review. Acantholysis was confirmed histologically in 146 of 162 cats (90%) and/or by cytological evaluation in 31 of 162 cats (19%). The exact age of onset of the disease was reported for 72 cats, with most cats being middle aged (median (mean): 7 (7.2) years; range: 0.25–16 years). In the remaining studies, age was reported as median and/or mean. In these studies, the median age of onset was 5 years (57 cats) [6] and 5.5 years (10 cats) [1], and the mean was 5.4 years (10 cats) [1], 6 years (8 cats) [4] and 7 years (15 cats) [7]. Females marginally out-numbered males (female-to-male ratio of 1.3). Cats affected with PF belonged to a variety of different breeds including Domestic short-haired (97/162; 60%), Siamese (13/162; 8%), Persian and Persian-crossbred (10/162; 6%), Burmese (7/162; 4%), Himalayan (5/162; 3%), Domestic medium-haired (5/162; 3%) and Domestic long-haired (5/162; 3%), Maine Coon (4/162; 3%), Birman (3/162; 2%), Russian blue (3/162; 2%), Tonkinese (2/162; 1%), and Bobtain cats (2/162; 1%) and one of each of the following breeds (American blue, Chinchilla, Cornish rex, Ragamuffin, Scottish fold and Somali cats).

In eight cats, a possible drug-association was proposed [6, 20, 22, 23, 27, 40]. In three of these eight cats, PF resolved spontaneously after the suspected drug(s) (cimetidine (1 cat), econazole/neomycin/triamcinolone/amoxicillin (1 cat), itraconazole/lime sulfur (1 cat)) were withdrawn; one cat experienced a flare up of disease when the offending drug (cimetidine) was restarted [6, 20, 23]. Another cat, in which doxycycline was suspected as the trigger, experienced a disease flare up after accidental reintroduction of the antibiotic. Disease control in this cat was achieved with an immunosuppressive treatment, which was eventually discontinued without further relapse [22]. One cat (methimazole suspected) had no available follow up, and the remaining three cats (cefovecin (1 cat), clindamycin/carprofen (1 cat) and ipodate (1 cat)) were successfully controlled with immunosuppressants, which were eventually withdrawn in two cats (cefovecin and clindamycin/carprofen) without reported relapse [6, 27, 40]. Re-exposure with the suspected drug(s) did not occur in the four latter cats.

Concurrent disease-association was proposed in three cats (thymoma (2) and leishmaniosis (1)) [28, 32, 35]. Thymoma removal and short-term immunosuppressive treatment resulted in a rapid DC in one cat [35]. In the other cat, DC was achieved with immunosuppressive treatment, but neither information about thymoma management nor complete drug withdrawal was available [32]. The cat with leishmaniosis received treatment for both leishmaniosis and PF; after reaching DC, the latter was later discontinued without further relapse [28].

Finally, one cat received standard vaccination shortly before the PF onset [38]. In this cat, a DC was achieved with immunosuppressive treatment, which was later completely stopped without a subsequent disease relapse.

Skin lesions in PF-affected cats consisted of pustules, erosions and crusts as expected based on the inclusion criteria. Due to the 34-year span of the selected publications and the inconsistency in the data reporting, not all information was available for each cat. The lesion distribution was symmetric in the majority of cats (127/131; 97%), and lesions usually affected two or more body regions (122/151; 81%). The two most commonly affected body regions were the face/head (122/145; 84%) and limbs (103/144; 72%); the most commonly affected skin sites being the pinnae (112/144; 78%) and claw folds (74/142; 52%) (Fig. 2). The majority of cats (83/114; 73%) were pruritic, and the degree of pruritus was noted in 13 of these 83 cats (mild: 8, moderate: 4, severe: 1). Non-dermatological signs such as the presence of systemic signs (in general) or specific comments regarding lethargy and fever were reported in 13/30 (43%), 33/72 (46%) and 18/67 (27%) cats, respectively.

### Treatment and outcome

#### Original case series

**Fig. 4:**
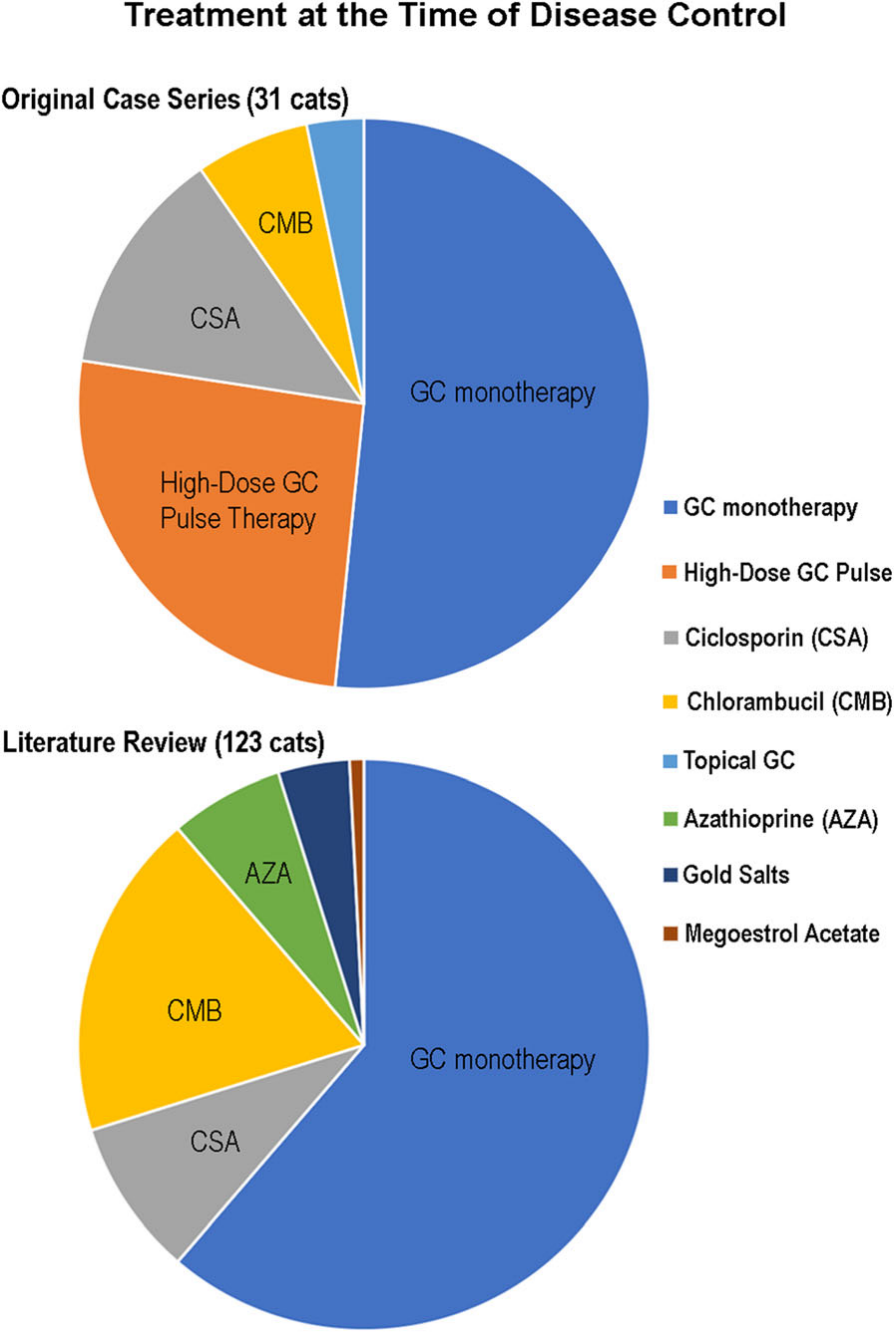
Pie charts of treatment regimens at the time of disease control

Treatment and outcome information was available for all 35 cats. The median time of follow-up was 15 months (mean: 23 months; range: 3.5–55 months). Disease control was achieved in 31 cats (89%) (Fig. 4). The median time to achieve DC was 22 days (mean: 37 days; range: 7–269 days).

Spontaneous remission was not observed in any cat. In approximately half of the cats, DC was achieved using glucocorticoid monotherapy (16/31; 52%) with standard dosages accepted in veterinary medicine [41]. Prednisolone was utilised most frequently (13/31; 42%), while prednisone, triamcinolone acetonide and dexamethasone were used in one cat each. The times to DC, median and mean dosages at the time of DC, where indicated, and the cumulative dosages of glucocorticoids received by the cats prior to DC (calculated as prednisolone equivalent per 5 kg cat [41, 42]) are listed in Table 1. The highest initial dosages of these drugs were identical to those used at the time of DC with the exception of triamcinolone acetonide (1 cat; initial dosage: 0.6 mg/kg/day).

**Table 1:** Original case series: Treatment details at the time of disease control

Treatment at the Time of Disease Control	# cats	% cats	Time to Disease Control (days)			Dosages of GC at the Time of Disease Control (mg/kg/day)			Cumulative Dose of GC up to DC (mg) (prednisolone equivalent for 5 kg cat)		
			Median	Mean	Range	Median	Mean	Range	Median	Mean	Range
Standard GC Monotherapy	16	52%	14	21	(7–50)				280	306	(35–640)
*prednisolone*	*13*	*42%*	*14*	*18*	*(8–31)*	*3*	*3*	*0.7–5.5*	*280*	*298*	*(35–600)*
*prednisone*	*1*	*3%*	*12*			*4.4*			*NA*		
*triamcinolone*	*1*	*3%*	*42*			*0.3*			*640*		
*dexamethasone*	*1*	*3%*	*50*			*0.1*			*72*		
Non-Steroidal Immunosuppressants as a Monotherapy or Combination Therapy	6	19%	58	105	(24–269)				433	412	(180–710)
*GC + ciclosporin*	*2*	*6%*	*149*	*149*	*(28–269)*	*6*	*7*	*(5–10)*	*490*	*490*	*(270–710)*
*GC + ciclosporin + topical GC*	*2*	*6%*	*52*	*52*	*(24–80)*				*449*	*449*	*(433–465)*
*GC + chlorambucil*	*1*	*3%*	*36*			*0.2 [every other day]*	*0.2 [every other day]*	*(0.15–0.3) [every other day]*	*180*		
*chlorambucil + topical GC*	*1*	*3%*	*192*								
Topical GC only	1	3%	28								

Cumulative dose of glucocorticoids up to the time of disease control was calculated as a prednisolone equivalent for 5 kg cat. Following estimated steroid equivalency conversions were used: dexamethasone and triamcinolone acetonide about seven times and methylprednisolone 1.3 times more potent than prednisolone [41, 42]. Due to the low bioavailability of prednisone in cats [94–96], the equivalency conversion between prednisone and prednisolone could not be determined and, therefore, the single case in which prednisone was used to induce disease control was excluded from the cumulative dose calculation

**Fig. 5:**
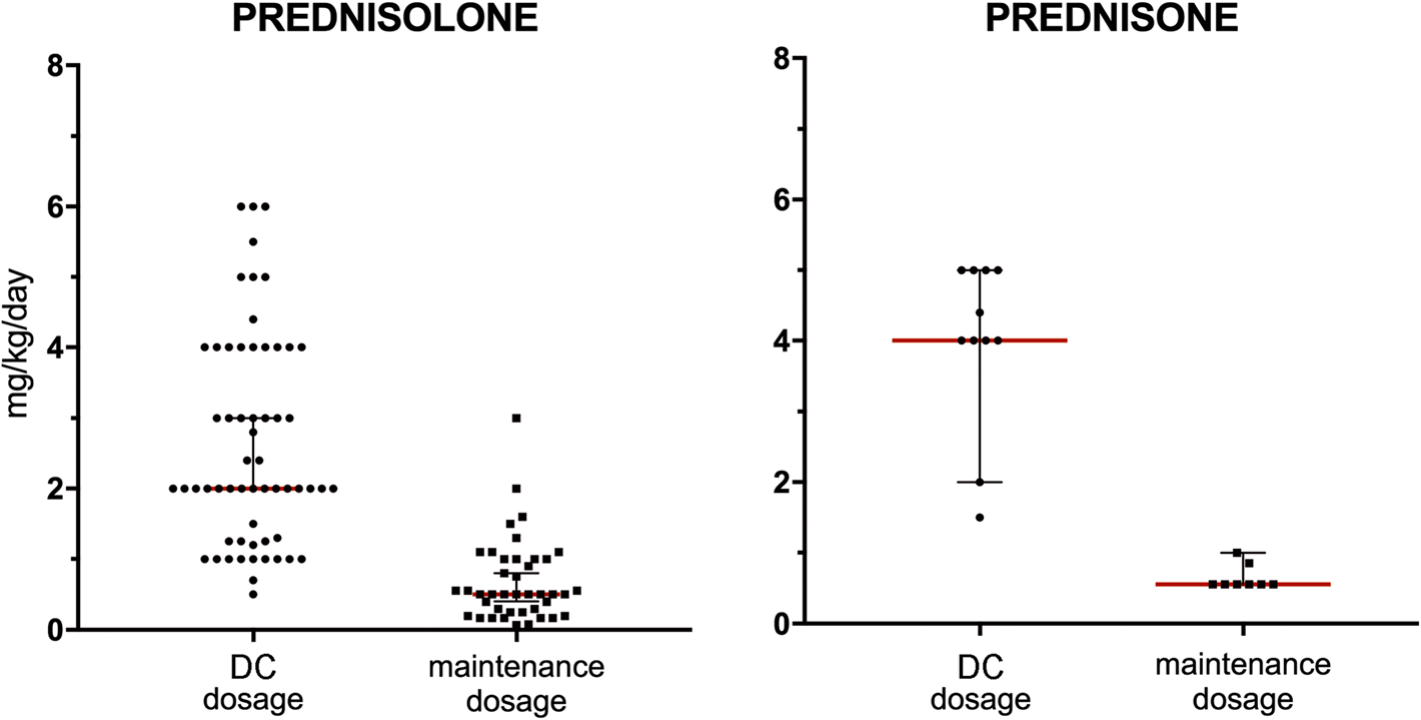
The maintenance dosages of oral glucocorticoids were significantly lower than those needed to induce disease control. A dot plot graph depicting daily dosages of individual cases; the horizontal red line indicates the median dosage and the vertical lines indicate 95% confidence interval (*p* values < 0.0001 for both prednisolone and prednisone dosages; Mann-Whitney test)

Twelve of the 31 cats (39%) received high-dose oral glucocorticoid pulse therapy using principles similar to those described in dogs (~ 10 mg/kg of prednisolone or prednisolone equivalent daily for three consecutive days, followed by a reduced dosage of a selected glucocorticoid (target: < 2 mg/kg/day of prednisolone or of its equivalent). Glucocorticoid pulse therapy could be repeated, at discretion of the clinician, if active lesions continued to appear after the dosage was tapered, but no more than one pulse per week was permitted [43]. For the pulse therapy, dexamethasone (9 cats; median: 1 mg/kg daily for 3 days [range: 0.8–1.2 mg/kg]), methylprednisolone (1 cat; 10 mg/kg), prednisolone (1 cat; 10 mg/kg) or triamcinolone acetonide (1 cat; 1 mg/kg) was utilised. Eight of the 12 cats (67%) reached DC with pulse therapy only; seven of them (58%) within 1 month. One (7 cats) to two (1 cat) pulse therapies were needed to induce DC in these cats (Table 2). In comparison, 14 of the 16 cats (88%) that received standard glucocorticoid monotherapy achieved DC within 1 month. The time to DC and the cumulative dosages of glucocorticoids received by cats prior to DC (calculated as prednisolone equivalent per 5 kg cat) are listed in Table 2. There was no statistically significant difference in the time to DC and the cumulative dose of glucocorticoids between the pulse and standard glucocorticoid monotherapy (*P* = *0.53* and *P* = *0.33*, respectively; Mann-Whitney U test) (Fig. 5).

**Table 2:** Original case series: Details of the high-dose pulse glucocorticoid therapy

High-Dose Glucocorticoid Pulse Therapy	# cats	% cats	Time to Disease Control			Cumulative Dose of Steroids			Number of Pulses		
			Median	Mean	Range	Median	Mean	Range	Median	Mean	Range
Total # of cats receiving high dose GC pulse therapy	12								1	2	(1–3)
Cats achieving DC within 4 weeks of the high dose GC pulse therapy	7	58%	14	14	(7–28)	165	198	(115–324)	1	1	1
Cats failing to reach DC within 4 weeks of the high dose GC pulse therapy *(eventually reaching DC with different TX*^a^*)*	3	25%	80	131	(43–269)	465	543	(465–710)	2	2	2
Cats treated with high dose GC pulse therapy that failed to reach DC *(including other treatment strategies)*	2	17%							3	3	3

Cumulative dose of glucocorticoids up to the time of disease control was calculated as a prednisolone equivalent for 5 kg cat. Following estimated steroid equivalency conversions were used: dexamethasone and triamcinolone acetonide about seven times and methylprednisolone 1.3 times more potent than prednisolone [41, 42]. Due to the low bioavailability of prednisone in cats [94–96], the equivalency conversion between prednisone and prednisolone could not be determined and, therefore, the single case in which prednisone was used to induce disease control was excluded from the cumulative dose calculation

^a^One of these cats achieved disease control with high-dose pulse glucocorticoid therapy within 43 days (after the second pulse). The remaining cats achieved disease control with other treatment regimens

Six of the 31 cats (19%) received non-steroidal immunosuppressive drugs such as ciclosporin or chlorambucil in combination with oral and/or topical glucocorticoids (Table 1). The times to DC, median and mean dosages of ciclosporin and chlorambucil at the time of DC, and the cumulative dosages of glucocorticoids (where indicated) received by the cats prior to DC (calculated as prednisolone equivalent per 5 kg cat) are listed in Table 1. The initial (highest) dosages of ciclosporin and chlorambucil were identical to those reported at the time of DC. Five cats received concurrent oral glucocorticoids at the time of DC including prednisolone (2 cats; median/mean dosage: 1 mg/kg/day [range: 0.5–1.5 mg/kg/day]), triamcinolone acetonide (2 cats; 0.2 mg/kg/day [range: 0.1–0.3 mg/kg/day]) or dexamethasone (1 cat; 0.1 mg/kg/day).

Topical glucocorticoids (most often 0.1% mometasone cream) were utilised at the time of DC in four cats. In one cat (3%), this was the sole treatment that led to DC within 28 days.

Five of the 30 cats in which DC was achieved with systemic treatment (17%) discontinued all systemic drugs without a disease flare up during the subsequent follow-up time period (range: 7 to 55 months). One cat required topical glucocorticoids to maintain DC. One cat was maintained with topical betamethasone ointment, but experienced a flare up of disease after 6 months of treatment at which point the owner elected euthanasia.

The remaining 24 cats (80%) were maintained on variety of systemic drugs with or without topical glucocorticoids (Table 3). Twelve of the 24 cats (50%) were maintained on reduced dosages of glucocorticoids, and 12 cats (50%) received non-steroidal immunosuppressants such as ciclosporin (7 cats) or chlorambucil (5 cats) as a monotherapy (5 cats), or in combination with glucocorticoids (7 cats). Topical glucocorticoids (mometasone or triamcinolone) were used concurrently with systemic treatment in four cats.

**Table 3:** Original case series: Maintenance treatment

Systemic Maintenance Treatment (24 cats total)		# cats	% cats	Dosages (mg/kg/day)		
				Median	Mean	Range
GC monotherapy		12	50%			
*prednisolone*		*7*	*29%*	*0.5*	*0.6*	*(0.2–1.3)*
*triamcinolone*		*2*	*8%*	*0.1*	*0.1*	*(0.05–0.1)*
*dexamethasone*		*3*	*13%*	*0.03*	*0.1*	*(0.03–0.1)*
Ciclosporin Monotherapy		*3*	*13%*	*5.3*	*4.8*	*(2.5–6.7)*
*ciclosporin + prednisolone*	*ciclosporin*	*3*	*13%*	*2.5*	*2*	*(0.7–2.5)*
	*prednisolone*			*0.1*	*0.4*	*(0.1–1)*
*ciclosporin + triamcinolone*	*ciclosporin*	*1*	*4%*	*2*		
	*triamcinolone*			*0.05*		
Chlorambucil Monotherapy		*2*	*8%*	*0.2*	*0.2*	*(0.15–0.2)*
*chlorambucil + prednisolone*	*chlorambucil*	*3*	*13%*	*0.1*	*0.1*	*no range detected*
	*prednisolone*			*0.5*	*0.7*	*(0.5–1)*

Disease flare ups were reported in the majority of cats (24/31; 77%). In 22 cats, disease flare ups coincided with a reduction of the drug dosage or treatment discontinuation. Two cats experienced intermittent flare ups of PF with the maintenance treatment.

Adverse effects related to treatment were reported in nine cats and included transient diabetes mellitus (3/9; 33%), mild to severe, undefined, upper respiratory tract disease (3/9; 33%), hepatopathy (3/9; 33%), polyuria/polydipsia (2/9; 22%), urinary tract infection (1/9; 11%) and bullous keratopathy (1/9; 11%). Two of the nine cats with adverse effects received one or two high-dose glucocorticoid pulse treatments (mild upper respiratory tract disease (2 cats) and transient diabetes mellitus (1 cat)), while the other seven cats received either standard glucocorticoid monotherapy (4 cats) or combination of glucocorticoids and non-steroidal immunosuppressants (3 cats).

Finally, 10 of the 35 cats diagnosed with PF (29%) died during the follow-up period. Two of the 10 cats (20%) died due to unrelated causes, and one (10%) died suddenly for unexplained reasons one year after the diagnosis confirmation. Four of 10 cats (40%) were euthanized due to the development of a non-dermatological disease such as chronic renal failure (2 cats) and neoplasia (2 cats). Two cats (20%) were euthanized after a disease flare up at which point they also suffered with a concurrent non-dermatological disease (diabetes mellitus (1 cat), upper respiratory infection (1 cat)), and one cat (10%) was euthanized due to the inability to rapidly control the recent flare up. The median time to death was 7 months (mean: 13 months; range: 6–29 months).

#### Comprehensive literature review

Treatment and outcome information was available for 140 of 162 cats (86%). The time of follow-up was reported in 93 cats with a median and mean of 13 and 20 months, respectively (range: 2–84 months). For additional 45 cats, the time of follow-up was reported as median only (9 months; range: 1–54 months) [6]. Disease control was achieved in 132 cats (94%). The treatment at the time of DC was known in 123 cats, and is summarized in Fig. 4 and Table 4. The time to DC was reported in 63 cats with median and mean being 21 and 28 days, respectively (range: 7–78 days). For an additional eight cats, the time to DC was reported as mean only (21 days; range: 14–30 days) [4]. Times to DC for individual treatment regimens are listed in Table 4. Three cats were reported to undergo spontaneous remission. In these three cats, an adverse drug reaction was suspected, and discontinuation of all therapeutics given just prior to the development of PF (cimetidine [1 cat], amoxicillin, triamcinolone, neomycin, enilconazole [1 cat] and itraconazole and sulfur dips [1 cat]) led to rapid resolution of all skin lesions [6, 20, 23].

**Table 4:** Comprehensive literature review: Treatment at the time of disease control

Treatment Regimen	Number of Cats (%) Treated	Time to Disease Control (days)			Drug Dosages at the Time of Disease Control (median, mean and range (mg/kg/day))					
		Median	Mean	Range	nonsteroidal drug			oral glucocorticoid		
					Median	Mean	Range	Median	Mean	Range
Oral Glucocorticoid Monotherapy (all)	76 (62%)	14	21	(7–70)						
*prednisolone*	*49 (40%)*	*14*	*21*	*(7–70)*				*2*	*2*	*(0.5–6)*
*triamcinolone*	*15 (12%)*	*nr*	*nr*	*nr*				*nr*	*nr*	*(0.6–2)*
*prednisone*	*10 (8%)*	*nr*	*nr*	*nr*				*4*	*4*	*(1.5–5)*
*dexamethasone*	*2 (2%)*	*nr*	*nr*	*nr*				*nr*	*nr*	*nr*
Non-Steroidal Immunosuppressants as a Monotherapy or Combination Therapy	47 (38%)									
Chlorambucil + Oral Glucocorticoid (all)	23 (19%)	32	36	(14–78)						
*chlorambucil + prednisone*	*9 (7%)*	*nr*	*nr*	*nr*	*nr*	*nr*	*nr*	*nr*	*nr*	*nr*
*chlorambucil + prednisolone*	*8 (7%)*	*32*	*36*	*(19–78)*	*0.1*	*0.2*	*(0.1–0.3)*	*3*	*4*	*(1.6–8)*
*chlorambucil + dexamethasone*	*4 (3%)*	*28*	*29*	*(14–45)*	*0.1*	*0.1*	*(0.1–0.2)*	*0.2*	*0.2*	*(0.1–0.4)*
*chlorambucil + triamcinolone*	*2 (2%)*	*nr*	*nr*	*nr*	*nr*	*nr*	*nr*	*nr*	*nr*	*nr*
Ciclosporin Monotherapy	2 (2%)	64	64	(55–73)	5	5	(5–5.6)			
Ciclosporin + Oral Glucocorticoid (all)	9 (7%)	37	41	(28–67)	5.1	5.3	(4.4–6.9)			
*ciclosporin + dexamethasone*	*3 (3%)*	*35*	*34*	*(28–38)*	*5.3*	*5.1*	*(4.4–5.5)*	*0.2*	*0.2*	*(0.1–0.2)*
*ciclosporin + prednisolone*	*2 (2%)*	*42*	*42*	*(33–50)*	*5.1*	*5.1*	*(5–5.2)*	*2.6*	*2.6*	*(2–3.1)*
*ciclosporin + triamcinolone*	*2 (2%)*	*36*			*6*	*6*	*(5–6.9)*	*0.6*	*0.6*	*(0.4–0.9)*
*ciclosporin + prednisone*	*1 (1%)*	*67*			*4.8*			*3*		
*ciclosporin + methylprednisolone*	*1 (1%)*	*42*			*nr*			*nr*		
Azathioprine + prednisone	8 (7%)	nr	21	(14–50)	1.1 mg/kg every other day			4.4 mg/kg/day		
Gold Salts + Oral Glucocorticoids	5 (4%)	nr			aurothioglucose: 0.5 mg/kg/week					

In some cats, disease control was induced with more than one treatment regimen; *nr* not reported

Glucocorticoid monotherapy was the most common treatment regimen administered at the time of DC (76/123; 62%) and used either prednisolone (49 cats), triamcinolone (15 cats), prednisone (10 cats), or dexamethasone (2 cats). Due to missing data, time to DC was reported for prednisolone only, and the median/mean dosages of glucocorticoids administered at the time of DC were calculated for prednisolone and prednisone only (Table 4). In the 15 cats that received triamcinolone, the dosage was reported by the authors as a range only (0.6–2 mg/kg/day) [6]. Topical glucocorticoids or tacrolimus were used concurrently with glucocorticoid monotherapy in four cats.

Oral glucocorticoids were frequently combined with chlorambucil (23/123; 19%) or ciclosporin (9/123; 9%), and topical glucocorticoids were used concurrently in seven of these cats (1% betamethasone cream or 0.015% triamcinolone spray). In two cats, ciclosporin was used alone to achieve DC. Time to DC and the median/mean daily dosages were reported in 10 chlorambucil- and 10 ciclosporin-receiving cats (Table 4).

Azathioprine combined with oral prednisone (8/123; 7%), gold salts (aurothioglucose or aurothiomalate) with oral glucocorticoids (5/123; 4%), and megoestrol acetate monotherapy (1/123; 1%) were used infrequently (Table 4).

Eighteen of the 129 cats (14%) in which DC was achieved discontinued all systemic drugs. The median and mean time to follow up for these cats was 18 and 23 months, respectively (range: 3.5–84 months; information available for 15 cats). In the remaining three cats, only the overall median time to follow up was known (9 months).

A maintenance regimen information was known for 98 of the 140 cats (70%) (Table 5). Oral glucocorticoid monotherapy was used most frequently (62/98; 63%), and included prednisolone (34/98; 35%), prednisone (12/98; 12%), triamcinolone (13/98; 13%) and dexamethasone (3/98; 3%). The median/mean daily dosages and ranges are reported in Table 5. In case of triamcinolone and dexamethasone, the maintenance dosages were reported only as a range (triamcinolone: 0.6–1 mg/kg every 1–7 days; dexamethasone: 1.5 mg/cat every 2–7 days) [6].

**Table 5:** Comprehensive literature review: Maintenance treatment

Systemic Maintenance Treatment (98 cats total)		# cats	% cats	Dosages (mg/kg/day)		
				Median	Mean	Range
GC monotherapy		62	63%			
*prednisolone*		*34*	*35%*	*0.5*	*0.6*	*(0.1–1.6)*
*prednisone*		*12*	*12%*	*0.6*	*0.6*	*(0.6–1)*
*triamcinolone*		*13*	*13%*	*nr*	*nr*	*0.6–1 every 1–7 days*
*dexamethasone*		*3*	*3%*	*nr*	*nr*	*1.5 mg/cat every 2–7 days*
glucocorticoids + doxycycline + niacinamide	doxycycline	4	4%	*nr*	*nr*	5–13 mg/kg once to twice daily
	niacinamide			*nr*	*nr*	25–44 mg/kg once to twice daily
	prednisolone/ prednisolone equivalent			0.4	0.4	(0.3–0.5)
Ciclosporin Monotherapy		6	6%	2.5	3.5	(1.7–6.7)
Ciclosporin (in combination with GC)		2	2%	4.2	4.2	(3.5–5)
Chlorambucil Monotherapy		7	7%	0.1	0.1	(0.1–0.2)
Chlorambucil (in combination with GC)		11	11%	0.1	0.1	(0.1–0.2)
Gold Salts monotherapy		3	3%	*nr*	*nr*	0.5–1 mg/kg once to twice weekly
Gold Salts (in combination with GC)		3	3%	*nr*	*nr*	

*nr* not reported

Oral glucocorticoids were combined with doxycycline and niacinamide (4/98; 4%), chlorambucil (11/98; 11%), ciclosporin (2/98; 2%) or gold salts (3/98; 3%) (Table 5). The three latter drugs were also able to maintain the PF in remission when used as a monotherapy (16/98; 16%) (Table 5).

Disease flare ups were reported in 55 of the 123 cats (45%) for which this information was provided, and they were most frequently associated with either a reduction of drug dosage or discontinuation of a treatment. In two cats, in which a drug-triggered PF was suspected, the disease relapsed after repeated administration of the offending drug (cimetidine (1 cat) and doxycycline (1 cat)) [20, 22].

Adverse effects related to treatment were reported in 39 of the 119 cats (33%) (cats with spontaneous remission of their disease, and those without relevant information were excluded). The most common adverse effects reported in cats receiving glucocorticoids included polyphagia and weight gain (8 cats), polyuria/polydipsia (7 cats), urinary tract infections (4 cats), hyperglycemia (2 cats), diarrhea or melena (3 cats), skin atrophy and skin fragility (2 cats), lethargy and anorexia (3 cats), pancreatitis (1 cat), and demodicosis (1 cat). Adverse effects reported in cats receiving chlorambucil alone or in combination with glucocorticoids included polyuria/polydipsia (4 cats), anorexia (3 cats), leukopenia (2 cats), thrombocytopenia (2 cats), anemia (1 cat) and increased liver enzymes (1 cat). Cats receiving ciclosporin alone or in combination with glucocorticoids developed disseminated mycobacteriosis (2 cats), diarrhea (1 cat) and hypertrichosis (1 cat). The use of azathioprine at 1.1 mg/kg every other day dosage was associated with leukopenia and neutropenia in the majority of cats (5/8 cats) receiving this medication. A corneal ulcer (1 cat) and skin abscess (1 cat) were observed in cats managed with gold salts and glucocorticoids, while the cat managed with megoestrol acetate developed demodicosis.

Nine of the 140 cats (6%) with known treatment and outcome died during the follow up period for variable reasons. One cat was euthanized immediately after the diagnosis confirmation and two shortly after due to treatment side effects or lack of DC. The rest of them developed non-dermatological diseases such as lymphoma (2 cats), disseminated mycobacteriosis (1 cat), pulmonary oedema and seizures (1 cat), cardiac arrest (1 cat) and severe gastrointestinal issues of unknown cause (1 cat). Most publications did not provide information about the time between the diagnosis confirmation and death.

## Discussion

The majority of publications about feline PF found between 1950 and 2016 only describe one to two cases. Our goal was to review the published literature and, with 35 original cases, provide a concise overview of what is currently known about feline PF.

Based on this review, pemphigus foliaceus affects middle aged cats (mean age of onset approximately 7 years), which is similar to dogs (4–6 years) and humans (40–60 years) [3, 44]. Domestic short-haired and Siamese cats were most commonly affected; however, a true breed predisposition could not be confirmed due to the nature of this study and the lack of population data for comparison. In dogs, a breed predisposition has been confirmed in Akitas and chow-chows, while in people, once endemic PF is excluded, no race/ethnicity predisposition has been observed [3, 44]. Although females marginally outnumbered males, sex predisposition could similarly not be confirmed. In canine and human PF, no sex predilection has been reported [3, 44].

Multiple triggers have been associated with development of PF in humans and dogs including drugs, pesticides/insecticides, neoplasia, immunization, infection, ultra-violet light, hormones and stress [3, 44–48]. Two cats with thymoma and concurrent PF have been published [32, 35]. Thymoma has been associated with a variety of autoimmune diseases in humans, cats and dogs, including different pemphigus variants [12, 49–52], and, therefore, it is possible that the PF in these cats was also related to the tumor’s aberrant effect on the immune system [53–55].

Variety of drugs have been associated with development of PF or PF-like disease in humans and dogs (reviewed in [45, 56]). This review identified eight cats in which drugs were proposed to be involved in the PF development, though only four of these cases would qualify as a probable drug reaction based on a retrospectively applied Naranjo drug reaction probability scale [6, 20, 22, 23]. Interestingly, one of these cats experienced a relapse of PF after reintroduction of doxycycline; a drug originally suspected to have been the trigger [22]. Considering the known anti-inflammatory properties of tetracycline antibiotics, and their use in managing some autoimmune skin diseases, including pemphigus, in humans and animals, this possible association is very unusual [57–59].

One cat with PF had concurrent leishmaniosis [28]. Similarly, PF and PF-like disease have been associated with canine and human leishmaniosis [60–62]. Whether the infection is the trigger for PF in these cases remains unknown. Interestingly, leishmaniosis and other vector-borne diseases had been hypothesized to play a role in human endemic PF in Brazil, but these have been recently replaced by a theory involving a sand fly salivary antigen molecular mimicry [62, 63].

In majority of cats, skin lesions involved more than one body region with head/face, claw folds and pawpads being the most frequently affected body sites (Figs. 2 and 3). Claw folds were the only affected body site in 11% of cats, and, therefore, PF should be considered as a relevant differential diagnosis in cats with erosive, exudative and/or crusting paronychia affecting the majority of digits. The periareolar region was affected in 10% of all cats for which this detail was provided, though there was difference between the numbers reported in the literature review (7%) and the original case series (20%). Similarly, the involvement of the perianal and/or perigenital region was more commonly mentioned in the original cases series (11%) than in the literature review (3%). These differences between the historically published literature and the original cases could be due to the failure to notice lesions in these sites or report these sites as a specific body region (e.g. periareolar region reported as a ventrum) or due to a true variation in incidence. Non-dermatological signs such as fever, lethargy and anorexia were reported in about half of all cats. In dogs, only two studies provide information on systemic signs, with one reporting one third of dogs to be lethargic [64], and the other mentioning that systemic signs usually accompanied only severe and more generalised disease [1]. In contrast, about half of dogs with an insecticide-triggered PF were reported to exhibit non-dermatological signs [46–48].

Feline PF has a good prognosis. The majority of cats (~ 90% on average) achieved DC in less than one month. This is in contrast to canine PF in which DC is achieved in a notably lower percentage of dogs (52% based on the largest case series) [65]. Additionally, some of the older studies reported “successful” treatment of PF in 53 and 88% of dogs [1, 64], but these studies did not define what the “successful” management meant in terms of the DC. Also in contrast to cats, dogs with PF require notably longer time to DC (based on the largest case series, the average times to DC with glucocorticoid monotherapy and with glucocorticoids and azathioprine combination were seven and 12 months, respectively) [65].

Glucocorticoids alone or in combination with non-steroidal immunosuppressants are the most commonly used drug class for the induction of DC; prednisolone monotherapy being one of the most common treatment strategies. According to the existing literature, the recommended dosages of prednisolone for feline PF vary from 2 to 6.6 mg/kg/day [1, 8, 66]. The analysis of the original cases and the literature review supports the effectiveness of dosages at the lower end of that recommended range. Information about the dosages of other oral glucocorticoids such as dexamethasone and triamcinolone was too limited to draw any meaningful conclusions.

A high-dose oral glucocorticoid pulse therapy following the principles described in PF-affected dogs [43] was used in 12 cats in an effort to achieve a faster DC and/or reduced overall cumulative dose of glucocorticoids. Interestingly, when the time to DC, the cumulative dose of glucocorticoids and the number of cats reaching DC within a month were compared between cats treated with high-dose glucocorticoid pulse therapy and standard glucocorticoid monotherapy, there was no obvious benefit of the former therapy. This perceived lack of added benefit of the pulse therapy could be explained by the observation that cats with PF respond to standard treatment protocols relatively rapidly. It is also possible that, due to the lower number of high-affinity glucocorticoid receptors in feline liver and skin compared to dogs [67], the dosage of prednisolone in the high-dose glucocorticoid pulse therapy for cats should have exceeded the 10 mg/kg dosage used in dogs [43]. Indeed, dosages of prednisolone as high as 8.8 mg/kg/day can be found in the literature and are recommended as part of standard immunosuppressive protocols (reviewed in [41]).

Ciclosporin and chlorambucil have been used by veterinary dermatologists to manage feline PF for years. Both drugs have been used also in canine PF [3], although a variable efficacy has been reported in case of ciclosporin [68–70]. Generally accepted dosages of ciclosporin and chlormabucil for management of autoimmune disorders in cats range between 5–10 mg/kg/day and 0.1–0.2 mg/kg/day or every other day, respectively [71]. These generally-accepted dosages were supported by those extrapolated from the original cases and the literature review here. The unusually long time to DC in the original case series cats treated with non-steroidal immunosuppressants (average time to DC: 105 days) might give an impression that cats receiving non-steroidal immunosuppressants alone or in combination with glucocorticoids require longer time to DC than those treated with glucocorticoid monotherapy. However, this longer time to DC with this treatment strategy was not observed in cats from the literature review (average time to DC: 41 days). The notably longer time to DC in cats from the former group is likely related to the standard of care used by clinicians contributing cases to the study. Indeed, the preferred treatment strategy at both institutions relies traditionally on glucocorticoid monotherapy, and non-steroidal drugs are only used when DC cannot be achieved in a timely fashion.

Most cats included in this study required long-term treatment and complete drug withdrawal with prolonged disease remission was reported only in the minority (17 and 14% in original cases and literature review, respectively). A similar outcome has been reported in dogs with naturally occurring PF in which complete treatment discontinuation was possible in 7 to 22% of cases (reviewed in [3]). The majority of cats requiring a long-term treatment received glucocorticoids at lower (anti-inflammatory) dosages than those used for induction of DC. Indeed, the maintenance dosages of prednisolone and prednisone were significantly lower than those at the time of DC (*p* value < 0.0001; Mann-Whitney test; Fig. 5). Statistical comparison for dexamethasone and triamcinolone dosages was not possible due to the low number of treated cats. Other treatments used for disease maintenance included ciclosporin or chlorambucil alone, or in combination with glucocorticoids. In most cats, the maintenance ciclosporin dosages were 25–50% lower than the initial dosages, though the range varied greatly (range: 0.7–6.7 mg/kg/day; every other day dosing was common). The maintenance dosages of chlorambucil varied between 0.1 and 0.2 mg/kg/day (every other day dosing was common). Topical glucocorticoids were useful in managing some cats alone or in combination with systemic treatment.

Despite the maintenance treatment, disease flare ups were frequent (77 and 45% in original cases and literature review, respectively), and usually followed a dose reduction or an attempt to discontinue treatment. In two cats with suspected drug reaction, a disease flare up followed shortly after re-introduction of the drug [20, 22].

Adverse effects related to the treatment were reported in one third of cats treated for PF. Diabetes mellitus, urinary tract infections and hepatopathy were the more severe signs usually reported in associated with glucocorticoids, while bone marrow suppression was more common in chlorambucil and azathioprine treated cats. The rapid onset of myelosuppression in the azathioprine treated cats was likely related to the used dosage (1.1 mg/kg every other day) and the lower level of thiopurine S-methyltransferase in this species, an enzyme responsible for the S-methylation of thiopurine drugs and inactivation of the cytotoxic 6-mercaptopurine [17]. Indeed, anecdotally, lower dosages (e.g. 0.3 mg/kg every other day) have been reported to be successful in managing other immune-mediated diseases [72].

Death or euthanasia directly related to the PF diagnosis (e.g. unwillingness to treat a cat with this condition) or a treatment failure (e.g. inability to induce DC, relapsing disease) or due to the occurrence of additional health issues, which might or might not have been related to the treatment, was reported in 10% of cats. This outcome appears to be markedly better than that reported for canine PF in which, based on one study, 42% of dogs were euthanized because of the lack of response to treatment, poor quality of life or due to treatment-associated adverse effects [73].

Finally, this retrospective case review has inherent limitations related to its predominantly clinical observational data, i.e. descriptions of skin lesion distributions, clinical signs, treatment responses, etc. Descriptions are only as detailed or accurate as reported, were made by different observers and were not collected by a standardized method.

In summary, feline PF is a pustular disease with secondary erosions and crusts, which usually predominate as a lesion type. The diagnosis of feline PF remains based on confirmation of subcorneal pustular disease, a rare lesion type in cats, and of its acantholytic nature. The majority of cats with PF exhibit lesions on the face and feet, though a subset of cats may exhibit lesions exclusively on the claw folds. Analysis of original cases herein suggests periareolar and perianal/perigenital area involvement to be more common than previously reported. Non-dermatological signs such as lethargy, fever and/or anorexia have been reported in more than half of cats with active disease. The prognosis of feline PF is good as the majority of cats rapidly achieve DC even with a simple immunosuppression protocol involving glucocorticoids monotherapy. However, well-designed studies comparing steroidal and non-steroidal treatment protocols are lacking. Most PF-affected cats require long-term treatment and, like other autoimmune disease, feline PF has a tendency to relapse spontaneously or with treatment changes. Owners should be informed and prepared for these circumstances, which may reduce the risk of euthanasia in case of disease relapse and improve treatment compliance.

## Abbreviations

DC: Disease control
PF: Pemphigus foliaceus
